# Real-time observation of cation exchange kinetics and dynamics at the muscovite-water interface

**DOI:** 10.1038/ncomms15826

**Published:** 2017-06-09

**Authors:** Sang Soo Lee, Paul Fenter, Kathryn L. Nagy, Neil C. Sturchio

**Affiliations:** 1 Chemical Sciences and Engineering Division, Argonne National Laboratory, 9700 South Cass Avenue, Argonne, Illinois 60439, USA; 2 Department of Earth and Environmental Sciences, University of Illinois at Chicago, 845 West Taylor Street, MC-186, Chicago, Illinois 60607, USA; 3 Department of Geological Sciences, University of Delaware, 255 Academy Street, Newark, Delaware 19716, USA

## Abstract

Ion exchange at charged solid–liquid interfaces is central to a broad range of chemical and transport phenomena. Real-time observations of adsorption/desorption at the molecular-scale elucidate exchange reaction pathways. Here we report temporal variation in the distribution of Rb^+^ species at the muscovite (001)–water interface during exchange with Na^+^. Time-resolved resonant anomalous X-ray reflectivity measurements at 25 °C reveal that Rb^+^ desorption occurs over several tens of seconds during which thermodynamically stable inner-sphere Rb^+^ slowly transforms to a less stable outer-sphere Rb^+^. In contrast, Rb^+^ adsorption is about twice as fast, proceeding from Rb^+^ in the bulk solution to the stable inner-sphere species. The Arrhenius plot of the adsorption/desorption rate constants measured from 9 to 55 °C shows that the pre-exponential factor for desorption is significantly smaller than that for adsorption, indicating that this reduced attempt frequency of cation detachment largely explains the slow cation exchange processes at the interface.

## Abstract (web summary)

Ion exchange at charged mineral-water interfaces is an important geochemical process, but a molecular-level understanding is still required. Here, the authors probe real-time variations of the interfacial ion exchange dynamics at the muscovite-water interface, providing a general picture of adsorbed ion coverage and speciation.

Charged solid–liquid interfaces are primary sites for a wide array of chemical reactions including ion adsorption and molecular uptake^1^,^2^,^3^,^4^,^5^, catalysis^6^,^7^,^8^,^9^,^10^ and energy storage^11^,^12^,^13^,^14^. Fundamental understanding of these processes depends on the ability to see changes in interfacial structure during reactions within the electrical double layer (EDL)^15^,^16^,^17^,^18^,^19^. Recent work^20^,^21^,^22^ demonstrated that the EDL structure at solid–water interfaces can be complex, having coexisting ions in different hydration states including inner-sphere (IS) complexes that adsorb at the surface in a partially solvated state and outer-sphere (OS) complexes that adsorb in a fully solvated state (Fig. 1a). Distinguishing these adsorbed species requires determination of the interfacial structure at the molecular scale. To date, knowledge of the dynamic variations in these adsorbed counterions largely relies on computational studies^4^,^23^,^24^,^25^. In contrast, experimental validations of ion dynamics at interfaces are scarce primarily because of difficulties in monitoring molecular-scale changes in the interfacial structure *in situ* and in real time. The reaction steps that control ion dynamics at interfaces, including reaction pathways that determine the overall reaction rates as well as energy barriers that separate distinct adsorption states within the pathways, are also not well defined.

Muscovite mica has a basal surface that is atomically flat with a known structural negative charge (1e^−^ per surface area of a unit cell, *A*_UC_=46.72 Å^2^, corresponding to −0.34 C m^−2^; ref. 26). Adsorption of simple alkali cations and associated changes of interfacial hydration at the muscovite (001)–water interface have been studied extensively using various approaches including surface force apparatus^27^,^28^,^29^,^30^, atomic force microscopy^31^,^32^, *in situ* X-ray surface scattering^22^,^33^,^34^,^35^,^36^ and computational simulations^34^,^37^,^38^. These studies unequivocally report that the reactivities of cations are closely related to their hydration. For example, a competitive adsorption isotherm between Rb^+^ and Na^+^ at the muscovite (001)–water interface revealed that the intrinsic adsorption constant (*K*°_ads_) for Na^+^ on the muscovite (001) surface is substantially lower than that for Rb^+^ (10^2.51±0.14^ versus 10^4.12±0.10^; refs 35, 39). The relatively weak adsorption for Na^+^ was interpreted to result from a larger energy cost for partial dehydration of more strongly hydrated Na^+^ compared to Rb^+^. The Gibbs free energy of hydration for Na^+^ is −365 kJ mol^−1^, and is significantly larger than −275 kJ mol^−1^ for Rb^+^ (ref. 40).

Differences in cation hydration can also influence adsorbed cation speciation at the interface. The distribution of Rb^+^ adsorbed at the muscovite (001)–water interface was determined to be a mixture of IS and OS complexes using *in situ* resonant anomalous X-ray reflectivity (RAXR)^22^ (Fig. 1). The fractional coverage of IS Rb^+^ was 85−90%, indicating that the majority of adsorbed Rb^+^ is in a partially solvated state, in which the muscovite surface completes the solvation shell. Dominant IS adsorption of Rb^+^ within the surface ditrigonal site of the muscovite surface also was reported from *in situ* atomic force microscopy^31^,^32^ and *in situ* crystal truncation rod (CTR) measurements^36^. In contrast, our understanding of the distribution of Na^+^ adsorbed at the muscovite (001)–water interface is still elusive. Unlike Rb^+^, whose distribution can be determined precisely using X-ray reflectivity (XR) and RAXR techniques^22^, the adsorbed distribution of Na^+^ ions is difficult to quantify by these techniques because of its small electron-density contrast with a water molecule^22^,^34^,^37^ and its X-ray absorption K-edge energy (1.071 keV) that is too low for *in situ* RAXR measurements. Instead, it was speculated that Na^+^ may adsorb to the muscovite surface at a greater distance from the surface (for example, as an OS complex) on the basis of its relatively weaker adsorption strength^35^ and stronger hydration strength^40^. Computational methods have been utilized to model the adsorbed structure of Na^+^ and the other alkali metal cations. However, the calculated structures depend strongly on the choice of force field. Molecular dynamics simulations with the Kawamura force field^37^ show that all alkali metal cations except Li^+^ adsorb as an IS complex in the ditrigonal cavity of the muscovite surface^34^,^37^. This observation contrasts with results using the Skipper force field^41^, which show preferential adsorption of cations on top of Al-substituted tetrahedra. The molecular dynamics simulations using the CLAYFF force field show that Na^+^ can form two types of IS species^38^, instead of one dominant species as observed by the other two calculations. Overall, experimental and computational studies indicate that it is well understood that Rb^+^ forms mostly an IS complex at the muscovite–water interface, whereas further observations are needed to understand the adsorbed structure of Na^+^ accurately.

Here we present experimental observations of the real-time evolution of adsorbed ion coverage and speciation during exchange reactions between Na^+^ and Rb^+^ at the muscovite (001)–water interface. Time-resolved RAXR measurements reveal both the significant difference between cation adsorption and desorption rates and asymmetric variations in adsorbed cation speciation at 25 °C. Rb^+^ adsorption is faster than Rb^+^ desorption, and appears to proceed directly to the dominant IS species. In contrast, Rb^+^ desorption is a slow stepwise process where thermodynamically stable IS Rb^+^ transforms to OS Rb^+^ before desorption from the interface. Real-time variations in Rb^+^ coverage at temperatures from 9 to 55 °C indicate that slow desorption is primarily controlled by a small pre-exponential factor attributed to the multiple paths each ion can take as it desorbs. Considering that the adsorption strength (as characterized by the intrinsic adsorption constant, *K*°_ads_) is determined by the relative magnitude between adsorption (*k*_ads_) and desorption (*k*_des_) rate constants, we conclude that the thermodynamic stability of these cation adsorbates is effectively determined by the rate of desorption, in particular the attempt frequency of ‘detachments' of ions from the muscovite–water interface.

## Results

### Cation exchange process and structure

Experiments were conducted using a freshly cleaved muscovite mica (001) surface in contact with two aqueous alkali metal salt solutions (30 mM NaCl and 3 mM RbCl). The NaCl concentration was chosen to be 10 times the RbCl concentration to mitigate the difference in adsorption constant between two cations. The intrinsic adsorption constant *K*°_ads_ for Rb^+^ on the muscovite (001) surface is about one order of magnitude higher than that for Na^+^ (10^4.12±0.10^ versus 10^2.51±0.14^; refs 35, 39).

**Figure 2:**
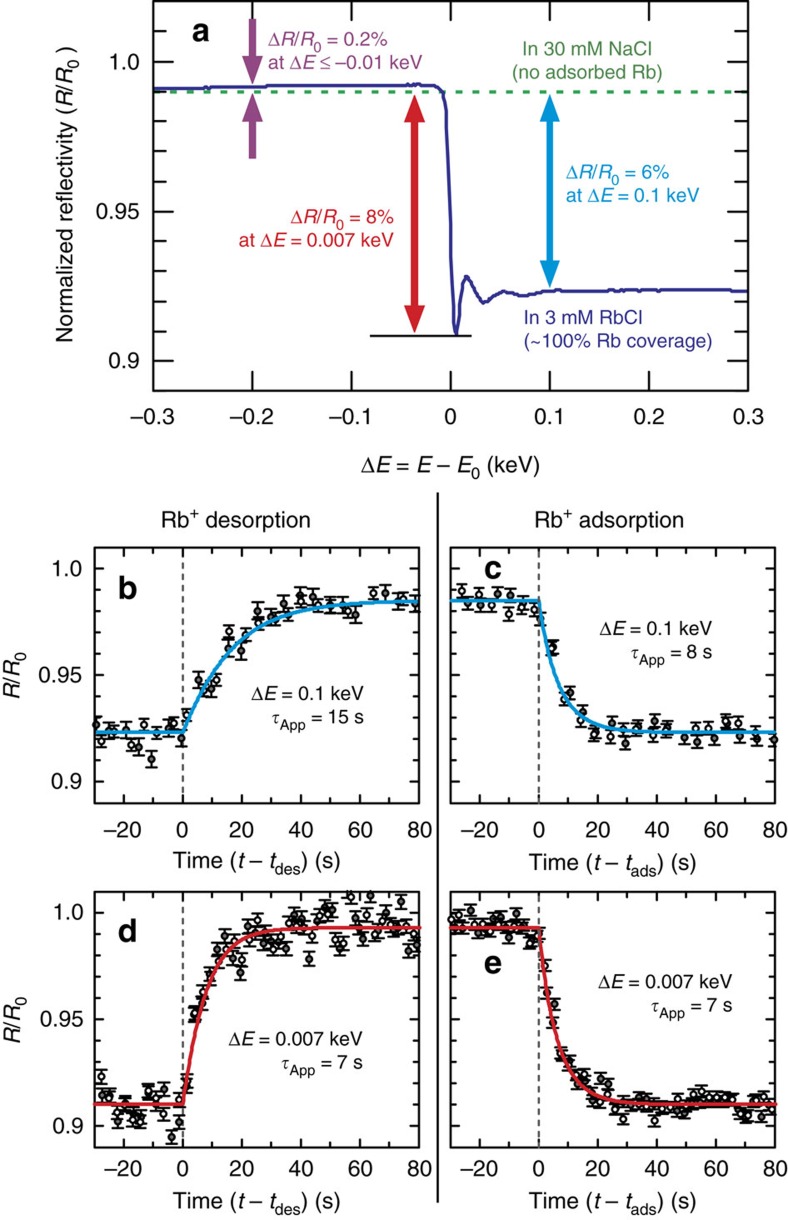
Real-time X-ray reflectivity during Rbsuper^+^/Na^+^ exchange at the muscovite mica (001)–water interface at a fixed *q*. (**a**) RAXR spectrum of muscovite (001) surface at *q*=0.488 Å^−1^ in 3 mM RbCl compared with that in 30 mM NaCl (ref. 22). The reflectivity (*R*) is normalized to the non-resonant reflectivity (*R*_0_) calculated using the best-fit model of the non-resonant XR data for the mica–RbCl system^22^. The purple, red and blue arrows indicate the relative changes at three different photon energies (*E*) near the X-ray absorption K-edge energy of Rb (*E*_0_=∼15.2 keV). (**b**–**e**) The temporal changes in reflectivity measured at Δ*E*=0.1 keV (and 0.007 keV) during Rb^+^ desorption and adsorption, including duplicate data (open and solid circles). One s.d. error bars are calculated on the basis of the counting statistics^43^. The solid curves are calculated from the best-fit first-order exchange equations where *τ*_App_ is the apparent time constant (Supplementary Note 3). In each plot, the time of injection of the exchanging solution (either *t*_des_ or *t*_ads_ for Rb^+^ desorption or adsorption, respectively) is indicated by a dashed vertical line.

Time-dependent changes in the interfacial structure during Rb^+^ and Na^+^ exchange were probed by using *in situ* time-resolved X-ray reflectivity (TXR). We utilized the resonant anomalous scattering phenomenon^42^ to obtain element-specific information through the variation in XR contrast as a function of photon energy. This is illustrated with a measured RAXR spectrum from the muscovite (001) surface in contact with a 3 mM RbCl solution^22^,^35^ at momentum transfer *q*=0.488 Å^−1^ (refs 35, 39) in Fig. 2a. This spectrum has a fractional modulation whose magnitude and shape are directly controlled by the coverage and average height of adsorbed Rb^+^, respectively^35^,^42^. Comparison of calculated RAXR spectra with and without Rb^+^ (that is, in 3 mM RbCl solution versus in 30 mM NaCl solution) shows strong contrast at *E*>*E*_o_, where *E*_o_=∼15.2 keV is the X-ray absorption K-edge energy of Rb. The maximum contrast (∼8%) can be obtained at Δ*E*=*E*−*E*_o_=0.007 keV where the RAXR spectrum in 3 mM RbCl has its minimum intensity.

Time-resolved XR data at Δ*E*=0.1 and 0.007 keV (Fig. 2b–e, respectively) were obtained while alternately flowing Rb^+^ and Na^+^ solutions across a muscovite (001) surface in a flow-through X-ray transmission cell^43^ (Supplementary Note 1) at 25 °C. The reflected X-ray intensities were fully recovered after each ion exchange cycle (Fig. 2b–e), indicating that the reactions are reversible. The rates of reaction were sensitive to both the reaction direction (for example, Rb^+^ adsorption versus desorption) and the photon energy where the XR data were measured. The apparent time constant (*τ*_App_) for Rb^+^ desorption at Δ*E*=0.1 keV was almost twice the value for Rb^+^ adsorption (15 versus 8 s, respectively, Supplementary Table 1), whereas *τ*_App_ values for both reaction directions are the same at a different photon energy, Δ*E*=0.007 keV. This difference in *τ*_App_ at two photon energies indicates that the shape of RAXR spectra may change during the exchange reaction (Supplementary Note 3). In particular, the spectral shape at this specific *q* is sensitive to the IS:OS coverage ratio^35^,^42^, implying variations in adsorbed Rb^+^ speciation during adsorption and desorption.

**Figure 3:**
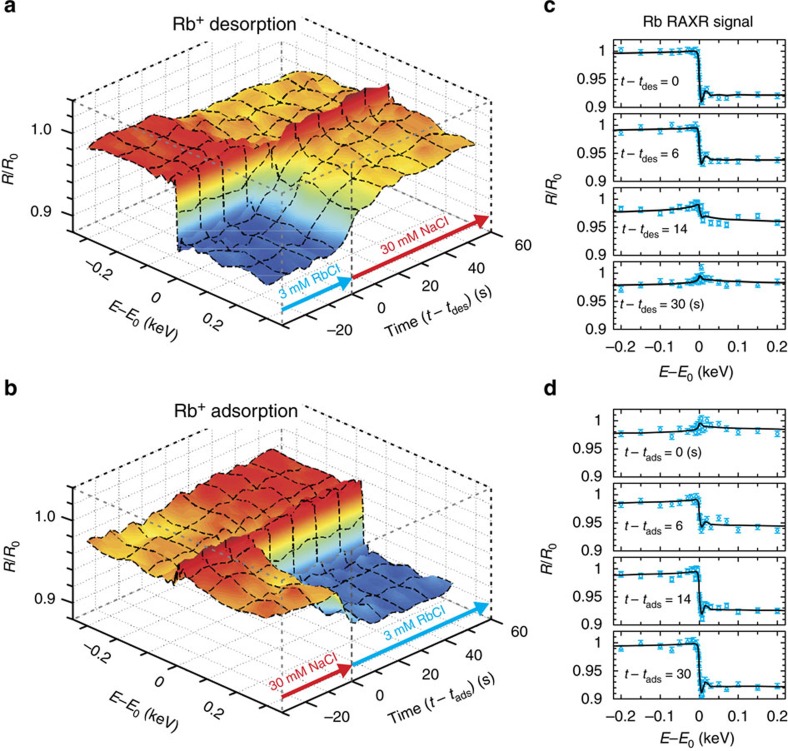
TRAXR at the muscovite mica (001)–water interface. X-ray reflectivity during Rb^+^ desorption (**a**) and adsorption (**b**) was measured as a function of photon energy (Δ*E*=*E−E*_o_) and reaction time (Δ*t*=*t*−*t*_des_ or *t*−*t*_ads_, respectively). (**c**,**d**) TRAXR spectra sampled at four different delay times after solution exchange during Rb^+^ desorption or adsorption. Error bars (s.d.) are derived on the basis of the counting statistics^43^.

To quantify the apparent differences in response time to the choice of photon energy, a series of XR data were obtained at 34 different photon energies near *E*_o_ (−0.3≤Δ*E*≤0.3 keV) as a function of reaction time (Fig. 3a,b). Sections parallel to the time axis are generally similar to those shown in Fig. 2, albeit with systematic energy-dependent variations in signal contrast as a function of photon energy. When the same data are viewed parallel to the energy axis, they correspond to a set of time-resolved RAXR (TRAXR) spectra, whose size and shape indicate the time-dependent variations in the Rb^+^ distribution at the interface (Supplementary Note 3).

Selected TRAXR spectra are compared in Fig. 3c,d to visualize differences in the time-dependent changes in the RAXR spectra between Rb^+^ desorption and adsorption. Compared with the spectrum at *t*−*t*_des_=0 s (where *t*_des_ corresponds to the time when Rb^+^ desorption started), the spectrum at *t*−*t*_des_=14 s has approximately half the RAXR magnitude near *E*_o_, indicating that about half of the Rb^+^ desorbed from the surface. In contrast, Rb^+^ adsorption was mostly complete in the spectrum at *t*−*t*_ads_=14 s (where *t*_ads_ corresponds to the time when Rb^+^ adsorption started), that is, the spectrum has almost the same magnitude as that at *t*−*t*_ads_=30 s. The spectra measured during Rb^+^ desorption (Fig. 3c) also show a notable change in shape at *t*−*t*_des_=14 and 30 s from the spectrum at *t*−*t*_des_=0 s, indicating that interfacial Rb^+^ distribution (that is, its speciation) changed during desorption^42^. On the other hand, the spectra (Fig. 3d) measured during Rb^+^ adsorption show almost no shape changes.

**Figure 4:**
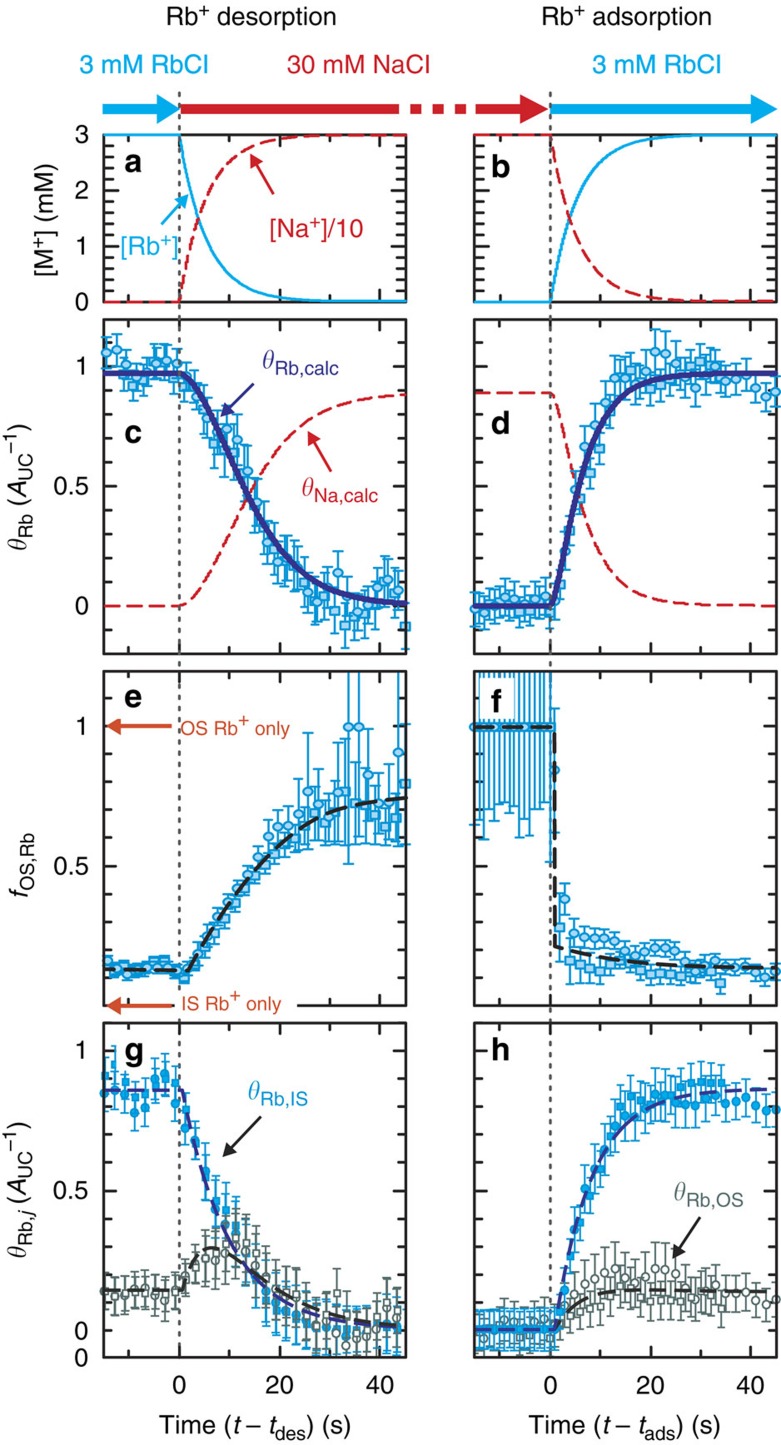
Temporal changes in the coverage and speciation of Rb^+^ adsorbed at the muscovite mica (001)–water interface during desorption and adsorption. (**a**,**b**) The calculated concentrations of Rb^+^ and Na^+^ in the experiment cell (see Supplementary Notes 1 and 2 for details). (**c**,**d**) Temporal variations in the total Rb^+^ coverage (*θ*_Rb_) during Rb^+^ desorption and adsorption. Each data set was duplicated (circle and square symbols). The thick solid blue lines represent the total Rb^+^ coverage (*θ*_Rb,calc_) calculated from the best-fit model (Table 1). The variations of Na^+^ coverage (*θ*_Na,calc_) calculated from the same model are also plotted in red dashed lines for comparison. (**e**,**f**) Temporal variations in the fractional coverage of OS Rb^+^ (*f*_OS,Rb_=*θ*_Rb,OS_/*θ*_Rb_) during desorption and adsorption. (**g**,**h**) Variations in the coverages of individual Rb^+^ species (*θ*_Rb,*j*_ where *j*=IS or OS) were calculated as *θ*_Rb,IS_=*θ*_Rb_ (1−*f*_OS,Rb_) and *θ*_Rb,OS_=*θ*_Rb_ *f*_OS,Rb_. The error bars represent s.d. uncertainties of the parameters derived from the least-squares analysis. The dashed lines are included to guide the eye.

These differences in the RAXR magnitude and shape indicate that temporal variations in the adsorbed Rb^+^ coverage and speciation depend on the reaction direction. To quantify this relationship, the time-dependent variations in the total Rb^+^ coverage and IS:OS coverage ratio were derived using model-independent RAXR analysis^35^,^42^ (Supplementary Note 4) as a function of Rb^+^ and Na^+^ concentrations in the cell during solution exchange (Fig. 4a,b and see Supplementary Notes 2 and 3 for details). Temporal variations in total Rb^+^ coverage show a significant difference in the reaction rate between desorption and adsorption (Fig. 4c,d). More than 90% of Rb^+^ desorbed in ∼25 s, whereas the same amount adsorbed within about half this time (∼14 s). This difference is correlated with the evolution of adsorbed Rb^+^ speciation during ion exchange. The fractional coverage of OS Rb^+^ (*f*_OS,Rb_) increased gradually from 0.15 (the equilibrium fractional speciation) to ∼0.7 during desorption. In contrast, a sudden change in *f*_OS,Rb_ was observed during Rb^+^ adsorption: *f*_OS,Rb_ rapidly decreased to ≤0.2 (close to the expected value of 0.1–0.15) within 5 s after injection of the RbCl solution (Fig. 4f), even though ∼20 s elapsed before the total Rb^+^ coverage reached its maximum (Fig. 4d).

The time-dependent changes in the IS and OS coverages of Rb^+^ were computed using *θ*_Rb_ and *f*_OS,Rb_. During Rb^+^ desorption (when the total ion coverage decreased monotonically), the coverage of OS Rb^+^ (*θ*_Rb,OS_) initially increased for ∼12 s after which it began to decrease gradually until almost all Rb^+^ desorbed from the surface (Fig. 4g). This result indicates that the redistribution rate of Rb^+^ between the IS and OS species is similar to the desorption rate of Rb^+^. This interpretation is consistent with the simulations shown in Supplementary Note 3. In contrast, the observed Rb^+^ speciation remains nearly unchanged during adsorption, with a value consistent with the equilibrium distribution (Fig. 4h), indicating that the rate for transformation from OS to IS Rb^+^ is significantly greater than the rate of adsorption.

### Determination of cation exchange kinetics

The temporal variations in the total coverages of adsorbed Rb^+^ and Na^+^ were characterized by intrinsic adsorption and desorption rate constants using equations for a two-component exchange reaction. The derivatives of the total coverages of each ion per *A*_UC_, *θ*_Rb_ and *θ*_Na_, with respect to time, are described by


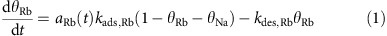



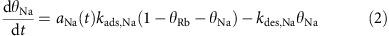


where *a*_Rb_(*t*) and *a*_Na_(*t*) are time-dependent ion activities calculated using the extended Debye–Hückel equation^44^ and cation concentrations in bulk solution (Fig. 4a,b and Supplementary Note 2), and *k*_ads,Rb_, *k*_des,Rb_, *k*_ads,Na_ and *k*_des,Na_ are the adsorption and desorption rate constants for Rb^+^ and Na^+^. These equations were solved numerically to determine the rate constants that best describe the real-time changes in Rb^+^ coverage. Although the Na^+^ coverages were not measured, their kinetic constants could be estimated indirectly on the basis of the effect on *θ*_Rb_ as described in equations (1 and 2).

There are similarities as well as differences in interfacial kinetics between the two cations. For both, the intrinsic rate constants for adsorption (*k*_ads_) are higher by several orders of magnitude than the intrinsic rate constants for desorption (*k*_des_; Table 1). Between these two cations, the intrinsic adsorption rate constant for Rb^+^ is more than 10 times higher than that for Na^+^, whereas the desorption rate constants for both cations are similar to each other. For both cations, the ratios *k*_ads_/*k*_des_ are similar to the intrinsic adsorption constants determined previously from the adsorption isotherm measurements at 25 °C (refs 35, 39), confirming the consistency between static and dynamic measurements^45^.

### Temperature-dependent cation exchange kinetics

Both the XR signals (at *q*=0.488 Å^−1^ and Δ*E*=0.007 or 0.1 keV; Fig. 2b–e) and the adsorbed Rb^+^ coverage values derived from TRAXR analyses (Fig. 4c,d) at 25 °C show monotonic variations as a function of reaction time (Supplementary Note 6). Using this relationship, the time-dependent variations in Rb^+^ coverage, referred to as ‘apparent Rb^+^ coverage' hereafter, can be estimated from the TXR data measured at these specific momentum transfer and photon energy. This method effectively bypasses the need for the full set of TRAXR data, and therefore allows us to measure the adsorption and desorption kinetics using the TXR data at selected photon energies (Δ*E*=0.007 or 0.1 keV) at four additional temperatures (9, 16, 40 and 55 °C).

**Figure 5:**
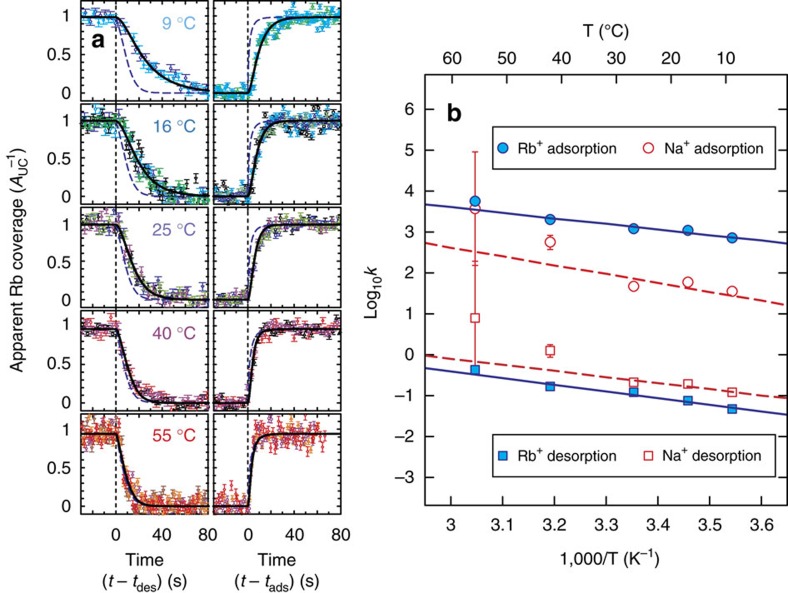
Exchange kinetics between Rb^+^ and Na^+^ adsorbed at the muscovite–water interface as a function of temperature. (**a**) Time-dependent change in apparent Rb^+^ coverage measured during Rb^+^ desorption (left) and adsorption (right) at 9, 16, 25, 40 and 55 °C. Each symbol with a different colour represents one independent data set. The s.d. error bars were calculated from the counting statistics^43^. Dotted vertical lines show the time when the solution exchange started (either *t*_des_ or *t*_ads_=0 for Rb^+^ desorption and adsorption, respectively). The solid lines show the Rb^+^ coverages calculated from the best-fit models using equations (1 and 2). The long-dashed lines are the equilibrium coverages calculated using the intrinsic adsorption constants^35^ and ion activities^44^, and the known solution exchange rates (Supplementary Note 2), and show the limit of the measurements for the fast reaction kinetics at 55 °C. (**b**) Arrhenius plot of the measured kinetic rate constants as a function of inverse temperature. The derived constants for Rb^+^ adsorption (solid circles) and desorption (solid squares) are shown with the best-fit lines (solid lines). The rate constants for Na^+^ adsorption (open circles) and desorption (open squares) obtained from this model, along with the best-fit trend lines (dashed lines), are shown for comparison.

The time-dependent variation in apparent Rb^+^ coverage (Fig. 5a) shows a strong dependence on temperature. As predicted from classical kinetic theories^45^, the reaction rates were slowest at the lowest temperature (9 °C), and increased with temperature. At 55 °C, the measured coverage variations almost overlap those calculated by assuming equilibration with the instantaneous solution composition (determined by the rate of solution exchange within the sample cell)^43^, indicating that the intrinsic reaction rates are difficult to determine precisely at this temperature.

The reaction rate constants at each temperature (Fig. 5b) were derived from the data using equations (1 and 2). Adsorption rate constants were about 4 orders of magnitude higher than desorption rate constants for Rb^+^ at all studied temperatures. The rate constants differed by 2 orders of magnitude for Na^+^. The rate constants for both Rb^+^ and Na^+^ generally show trends following a classical Arrhenius law^45^, although deviations from this linearity were observed for the rate constants of Na^+^ at higher temperatures where the measurements are less precise.

The derived rate constants were fit to the Arrhenius equation


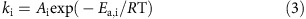


where *k*_i_, *A*_i_ and *E*_a,i_ are the intrinsic rate constant, Arrhenius pre-exponential factor (an attempt frequency for a reaction) and activation energy for reaction i; *R* is the gas constant; and T is the temperature. The activation energies for Rb^+^ adsorption and desorption are similar (26 and 31 kJ mol^−1^, respectively, Table 2). However, the pre-exponential factor for adsorption is over three orders of magnitude greater than for desorption. For Na^+^, the Arrhenius fit showed good agreement with the low-temperature data, but not the higher temperature rate constants determined with larger uncertainties. The *E*_a,ads_ value for Na^+^ was ∼14 kJ mol^−1^ higher than for Rb^+^, and the *A*_ads_ value was ∼20 times higher than that for Rb^+^. Both *E*_a,des_ and *A*_des_ values for Na^+^ were similar to those for Rb^+^, consistent with the similar *k*_des_ values observed for these two cations at 25 °C (Table 1).

## Discussion

**Figure 6:**
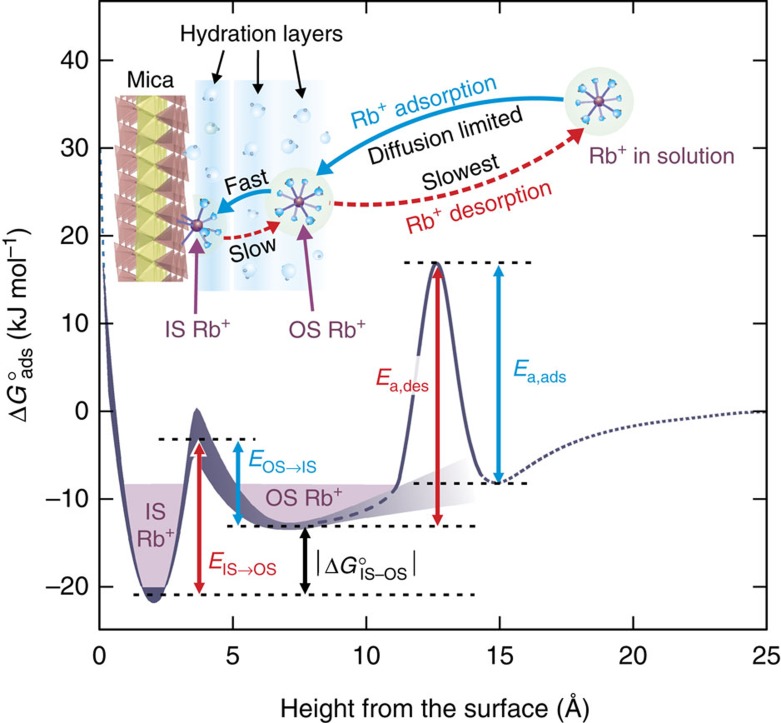
Free energy profile for Rb^+^ adsorbed at the muscovite mica (001)–water interface. The free energy profile (Δ*G*°_ads_) is computed as a function of height (*z*) up to ∼10 Å using the electron-density profile of adsorbed Rb^+^ reported previously^22^ (Supplementary Note 5). The thickness of the line corresponds to one s.d. of uncertainty^65^ of the free energy in units of kJ mol^−1^. The Δ*G*°_ads_ for *z*→∞ was set to 0 (Supplementary Note 5). The activation energies for the transition from IS to OS Rb^+^ and from OS to IS Rb^+^ are labelled *E*_IS→OS_ and *E*_OS→IS_, respectively. Δ*G*°_IS–OS_ is the difference in free energy of adsorption between IS and OS Rb^+^. The activation energies for Rb^+^ desorption and adsorption are labelled as *E*_a,des_ and *E*_a,ads_, respectively. The top schematic shows the stepwise pathways for adsorption and desorption of Rb^+^ (indicated with blue and red arrows, respectively) at the interface.

Time-resolved RAXR reveals that Rb^+^ desorption is a slow stepwise reaction in which the thermodynamically stable IS Rb^+^ transforms to the less stable OS Rb^+^ before it desorbs from the interface. In contrast, the adsorption of Rb^+^ proceeds rapidly to the stable adsorption mode in which Rb^+^ adsorbs dominantly as an IS species. These asymmetric variations on Rb^+^ speciation with reaction direction can be understood in the context of the free energy profile of interfacial Rb^+^ (Fig. 6). The relative free energies of adsorption for the IS and OS species, the widths of the free energy minima and a lower limit for the energy barrier for the transition between the IS and OS states are obtained from the electron-density profile of Rb^+^ adsorbed on the muscovite surface derived from previous RAXR measurements^22^ (see Supplementary Note 5 for details). The free energy profile for IS Rb^+^ is deeper than OS Rb^+^, reflecting thermodynamic stability of IS Rb^+^ over OS Rb^+^. Because IS Rb^+^ is more stable than OS Rb^+^ by ∼6 kJ mol^−1^ (corresponding to |Δ*G*°_IS−OS_| in Fig. 6), the energy barrier for the transition from IS to OS for Rb^+^ during desorption (*E*_IS→OS_≈18 kJ mol^−1^) is higher than the energy barrier for the transition from OS to IS during adsorption (*E*_OS→IS_≈12 kJ mol^−1^; Fig. 6).

The Arrhenius pre-exponential factors for cation adsorption to muscovite mica agree with predictions from classical ion kinetic theory for transport of solutes in a dilute solution^46^,^47^. The effective collision frequency (*J*_ion_) of a solute ion within the muscovite surface unit cell area can be calculated as


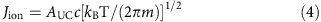


where *c* is the bulk ion concentration (atom Å^−3^), *k*_B_ is the Boltzmann constant (J K^−1^), T is temperature (K) and *m* is ion mass (=standard atomic weight/*N*_A_ where *N*_A_ is Avogadro's number). For Rb^+^ in a 3 mM RbCl solution, *J*_ion_=5 × 10^7^ s^−1^, a value similar to that fit to the XR data, *A*_ads_=8.5 × 10^7^ s^−1^ (Table 2). For Na^+^ in a 30 mM NaCl solution, *J*_ion_=∼1 × 10^9^ s^−1^, also in good agreement with *A*_ads_=2 × 10^9^ s^−1^. This agreement for both cations indicates that the attempt frequency for the counterion adsorption reaction on this charged muscovite surface is controlled by bulk ion diffusion and confirms the accuracy of our analysis.

In contrast, the pre-exponential factors for desorption, ∼3 × 10^4^ for Rb^+^ and ∼5 × 10^4^ s^−1^ for Na^+^, were significantly smaller than the values (≥10^10 ^s^−1^; ref. 48) expected for an elementary desorption process. For a simple interfacial system, such as a metal–vacuum interface, the attempt frequency for desorption is reported to be similar to the vibrational frequency of the adsorbate^48^,^49^. The observed small pre-exponential factors, therefore, indicate that desorption of a cation from the muscovite–water interface is intrinsically complex. It is proposed that cations undergo multiple steps in the process of detaching from the interface, for example, transformation from IS to OS complexes. Cations adsorbed at the muscovite surface are reported to have strong correlations with surrounding water molecules and even with neighbouring ions at higher adsorbed coverages^31^,^32^. Desorption of cations from the interface likely involves the correlated motion of multiple interfacial species. The motion of interfacial species can also be dampened by the increased viscosity of water near the charged surface. Computational studies^21^,^50^,^51^ showed that interfacial water near charged surfaces is more viscous than bulk water, as a result of the formation of well-ordered hydration layers near the surface^52^,^53^,^54^,^55^. Surface force apparatus^30^ and *in situ* XR measurements^22^,^56^ also confirmed experimentally the presence of structured interfacial water having a characteristic oscillation in density extending up to 10–15 Å from the muscovite surface.

The energy associated with disruption and reconfiguration of the interfacial hydration network can explain the magnitude of the observed activation energies for cation adsorption and desorption. In this context, the similarity of the activation energies for adsorption and desorption with the standard-state Gibbs free energy for the surface hydration of muscovite (between 10 and 20 kJ mol^−1^; ref. 57) is expected. However, the observed *E*_a_ values (26–40 kJ mol^−1^; Table 2) are higher, indicating that additional energy is required to explain the difference. We note that adsorption and desorption processes also involve the (partial) disruption of the hydration shells around the cations. The relative differences in activation energies for Na^+^ and Rb^+^ can be understood by the relative differences in the ion hydration energies, that is, there is a larger barrier to adsorption for the more strongly hydrated Na^+^. However, the activation energies are substantially smaller in magnitude than the hydration free energies for the cations (−365 kJ mol^−1^ for Na^+^ and −275 kJ mol^−1^ for Rb^+^; ref. 40), indicating that adsorption and desorption likely involves only a small change of the coordinating structure of the cations, for example, via ligand exchange between water molecules in the ion hydration shell and oxygen atoms on the muscovite surface.

Our measurements, to the best of our knowledge, are the first to monitor simultaneously the structure, kinetics and dynamics at a solid–water interface with molecular-scale sensitivity. Exchange kinetics of monovalent cations at the negatively charged muscovite surface were described using a simple kinetic model for the primary rate-limiting step reaction to provide a general understanding of how adsorbed cations change their coverage and speciation at this idealized interfacial system. These processes may be more complex when viewed with a better time resolution and over an extended range of temperature. Properties of adsorbates can also influence the interfacial thermodynamics and kinetics, especially for multivalent cations (for example, Sr^2+^ or Y^3+^)^39^,^58^ whose adsorption can be modified by ion–ion correlations^59^,^60^. In other interfacial systems where ions adsorb chemically to surface functional groups, we expect that larger activation energies will be observed when adsorption/desorption reactions involve the creation and breakage of chemical bonds (for example, on oxide surfaces)^1^,^61^.

These real-time observations of interfacial dynamics during cation exchange can have wide applicability for understanding reactions at various liquid–solid interfaces. For example, they can provide a fundamental basis for predictive models that describe the transport of dissolved species (for example, nutrients, contaminants) in natural systems or the effectiveness of water purification methods^62^,^63^ where ion exchange is a rate-limiting step. The quantitative description of ion exchange at the molecular scale can be used to understand and/or design chemical processes, such as nucleation, growth and dissolution of solids for which adsorption and desorption of constituent ions are the elementary steps, or to investigate the fundamental limitations of charge and discharge rates in supercapacitors^11^,^64^ where the mechanism of energy storage involves adsorption and desorption processes.

## Methods

### Sample preparation

Experimental solutions were prepared by dissolving high-purity rubidium chloride or sodium chloride in deionized water (18.2 MΩ cm). The final concentrations were 3 and 30 mM for Rb^+^ and Na^+^, respectively. In each solution, the cation coverage on the mica surface is calculated to be 97% and 90%, respectively, using the intrinsic adsorption constants for Rb^+^ and Na^+^ (refs 35, 39). The solution pH was adjusted to 7.5 using a small amount of 0.1 M NaOH to reduce the competition from hydronium^39^. A gem-quality muscovite crystal from Asheville Schoonmaker Mica Company was prepared with dimensions of 2.5 × 25 × 0.2 mm^3^. The crystal was cleaved using tape, rinsed with deionized water and transferred to an *in situ* flow-through X-ray transmission cell (Supplementary Fig. 1)^43^.

### X-ray measurements

X-ray experiments were conducted at beamlines 6-ID-B and 33-ID-D, Advanced Photon Source at the Argonne National Laboratory. The incident X-ray beam was focused vertically using a Kirkpatrick-Baez mirror to have a beam size of 0.02–0.05 mm (vertical) × 0.5–1.0 mm (horizontal) at the sample location with a flux of ∼5 × 10^11^ photons s^−1^. The X-ray beam reflected at the mica–solution interface was collected using an X-ray charged-couple device detector as a function of time at a fixed momentum transfer *q*=0.488 Å^−1^. This scattering condition was chosen to enhance the sensitivity of XR to the total coverage and average height of Rb^+^ adsorbed at the interface^35^,^39^. TRAXR data were obtained from a series of TXR data. The reflectivity at a given time was extracted from each TXR curve by linear interpolation, and the points calculated at 34 different photon energies near *E*_o_ at the same time were combined to yield a TRAXR spectrum at this given time. Each spectrum was analysed using model-independent RAXR analyses^42^ following the procedure described previously^35^.

### Data availability

The data that support the findings of this study are available from the corresponding authors on request.

## Additional information

**How to cite this article:** Lee, S. S. *et al*. Real-time observation of cation exchange kinetics and dynamics at the muscovite-water interface. *Nat. Commun.* **8,** 15826 doi: 10.1038/ncomms15826 (2017).

**Publisher's note:** Springer Nature remains neutral with regard to jurisdictional claims in published maps and institutional affiliations.

## Supplementary Material

Supplementary Information Supplementary Figures, Supplementary Tables, Supplementary Notes and Supplementary References

## Figures and Tables

**Figure 1:**
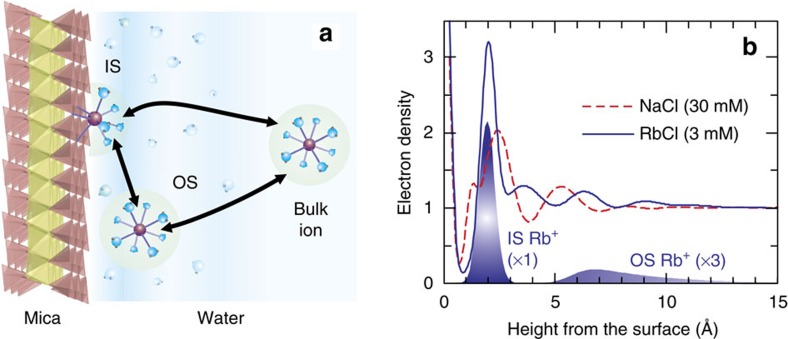
Monovalent cation exchange and interfacial structure at the muscovite mica (001)–water interface. (**a**) Schematic of the EDL at the muscovite–water interface. Monovalent cations adsorb as a combination of partially hydrated IS and fully-hydrated OS complexes, with several potential pathways for exchange between the adsorbed and bulk solution species. (**b**) Interfacial structure between the mica (001) surface and 30 mM NaCl versus 3 mM RbCl solutions^22^,^56^. The total electron-density profiles determined by high-resolution XR are shown for the two solutions. The distribution of IS and OS Rb^+^ determined by RAXR is shown in shaded areas. The height is referenced to the average height of oxygen in the mica surface. The distribution of OS Rb^+^ is scaled vertically by a multiplicative factor of 3 for clarity.

**Table 1:** Kinetic and thermodynamic properties of adsorption and desorption of Rb^+^ and Na^+^ at the muscovite mica (001)–water interface at 25 °C.

**Cations**	**Reaction**	***k* (s^−1^)**	***k*_ads_/*k*_des_**	***K*°_ads_**	**Δ*G*°_hyd_ (kJ mol^−1^)**
Rb^+^	Ads	2.1 (±0.6) × 10^3^	10^4.2(±0.2)^	10^4.12 (±0.10)^	−275
	Des	0.14 (±0.01)			
Na^+^	Ads	1.3 (±0.5) × 10^2^	10^2.7(±0.3)^	10^2.51 (±0.14)^	−365
	Des	0.25 (±0.03)			

Ads, adsorption; Des, desorption; Δ*G*°_hyd_, standard-state Gibbs free energy of hydration^40^; *k*, intrinsic rate constant for ads and des at 25 °C; *K*°_ads_, intrinsic adsorption constant in the standard state^35^.

The numbers in parentheses are one s.d. uncertainties of the parameters.

**Table 2:** Arrhenius analyses of the temperature-dependent real-time XR data during Rb^+^ and Na^+^ exchange at the muscovite mica (001)–water interface.

**Cations**	**Reaction**	***E*_a_ (kJ mol^−1^)**	***A* (s^−1^)**
Rb^+^	Ads	26.1 (±0.3)	8.5 (±1.1) × 10^7^
	Des	30.9 (±0.3)	3.0 (±0.4) × 10^4^
Na^+^	Ads	40.2 (±1.2)	2 (±1) × 10^9^
	Des	29.7 (±0.5)	5 (±1) × 10^4^

*A*, Arrhenius pre-exponential factor; *E*_a_, activation energy; XR, X-ray reflectivity.

Reaction: adsorption and desorption.

The numbers in the parentheses are one s.d. uncertainties of the parameters.
