# Supplementary material for: Real-time observation of cation exchange kinetics and dynamics at the muscovite-water interface
Source: Nat Commun. 2017 Jun 9;8:15826. doi: 10.1038/ncomms15826 (PMC5472772; doi:10.1038/ncomms15826)
Supplement: Supplementary Information — Supplementary Figures, Supplementary Tables, Supplementary Notes and Supplementary References [file ncomms15826-s1.pdf]

Supplementary Note 1: In-situ flow-through X-ray transmission cell

Details of the cell geometry and its performance for X-ray scattering measurements are described elsewhere[1]. The cell (Supplementary Fig. 1) was attached with two automated syringe pumps to which two syringes containing 3 mM RbCl and 30 mM NaCl solutions were mounted. Flow rates of these two pumps were changed to exchange solutions in the cell. For each exchange sequence, a new solution was injected initially with a flow rate of 8 mL min^{-1} for 30–60 s and then with a slower rate (either 0.5 or 1 mL min^{-1}).

The solution exchange rate in the cell was calculated on the basis of temporal variations in X-ray transmission measured at 0.1 keV above the K-absorption edge of Rb (E_o) during exchange between deionized water (DIW) and 0.1 M RbCl solution. The data were collected at 9 locations about 20 μm (i.e., ~vertical size of the X-ray beam, see Methods for details) above the mica surface. Temporal variations in X-ray transmission ($T = I_T/I_o$ where I_T and I_o are transmitted and incident beam intensities, respectively) through the cell can be expressed as

$$T(t) = T_0 \exp[-\mu_{Rb}(t)] \qquad (1)$$

where T_0 is the X-ray transmission through the cell when Rb^+ concentration (c_{Rb}) = 0, and $\mu_{Rb}(t)$ is the X-ray attenuation factor by Rb^+ in the cell as a function of time, which is approximately proportional to c_{Rb}. From equation (1), time-dependent variations in c_{Rb} can be written as

$$c_{Rb}(t) = p(\log T(t) - \log T_w) \qquad (2)$$

where p is a constant. The exchange data from RbCl to DIW and from DIW to RbCl (Supplementary Figs. 2a and 2b, respectively) were fit to exponential decay functions expressed as

$$c_{Rb}(t) = p(\log T(t) - \log T_w) = c_{Rb,max}\,[1-\exp(-t/\tau_{cell})] \qquad (3)$$

$$c_{Rb}(t) = p(\log T(t) - \log T_w) = c_{Rb,max}\exp(-t/\tau_{cell}) \qquad (4)$$

using the maximum Rb concentration ($c_{Rb,max}$ = 0.1 M) and the time constant (τ_{cell}) for the solution exchange in the sample cell. The derived τ_{cell} value for all datasets (18 total) was 5.3 ± 0.3 s.

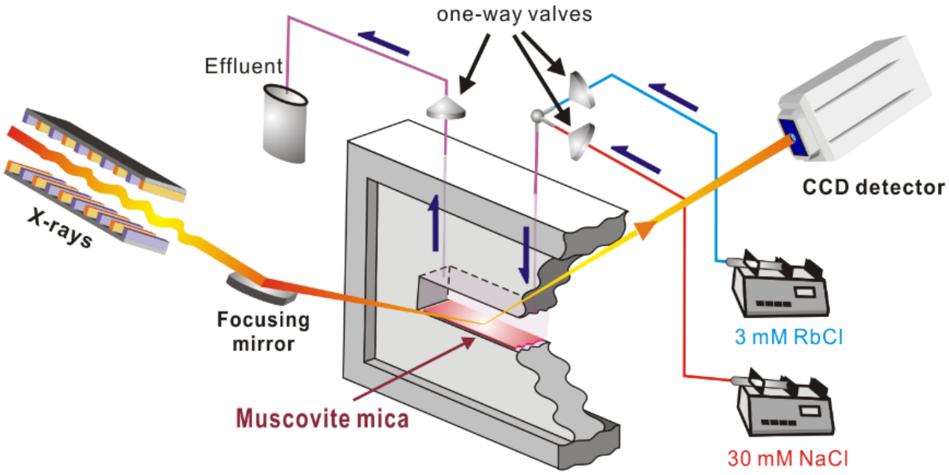

Supplementary Figure 1. Schematic of the in-situ flow-through X-ray transmission cell[1].

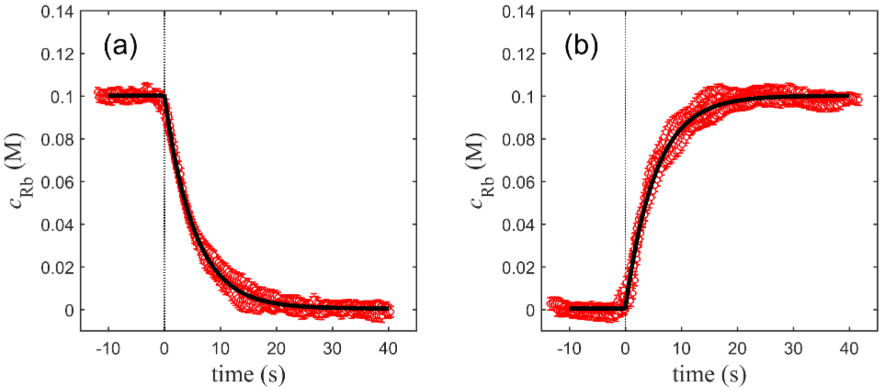

Supplementary Figure 2. Solution exchange rate measurements at the muscovite (001) – water interface. The concentration of Rb^+ was calculated from X-ray transmission (at $\Delta E = E - E_o = 0.1$ keV) measured through an in-situ X-ray transmission cell using equations (2–4). The data were collected during exchange from 0.1 M RbCl to DIW (a) and exchange from DIW to 0.1 M RbCl (b) with a flow rate of 8 mL min^{-1}. The one standard deviation (s.d.) error bars were obtained using the counting statistics.

Supplementary Note 2: Time-dependent cation coverages

The equilibrium coverages of Rb^+ and Na^+ (Figs. 4a–b), the coverages expected when the interface is fully equilibrated with a solution containing both Rb^+ and Na^+ at time t, were calculated using Langmuir isotherms[2] as

$$\theta_{Rb,eq}(t) = a_{Rb}(t)\, K^{o}_{ads,Rb}/(1+a_{Rb}(t)\, K^{o}_{ads,Rb}+a_{Na}(t)\, K^{o}_{ads,Na}) \quad (5)$$

$$\theta_{Na,eq}(t) = a_{Na}(t)\, K^{o}_{ads,Na}/(1+a_{Rb}(t)\, K^{o}_{ads,Rb}+a_{Na}(t)\, K^{o}_{ads,Na}) \quad (6)$$

where $K^{o}_{ads,Rb}$ and $K^{o}_{ads,Na}$ are the intrinsic adsorption constants of Rb^+ and Na^+ at 25°C, respectively[3,4]. The values $a_{Rb}(t)$ and $a_{Na}(t)$ are the activities of Rb^+ and Na^+ at time t, and were calculated using the Rb^+ and Na^+ concentrations at t ($c_{Rb}(t)$ and $c_{Na}(t)$, respectively) computed from equations (3 and 4) using the time constant (τ_{cell} = 5.3 sec) for the solution exchange in the sample cell (Supplementary Note 1). The extended Debye-Hückel theory[2] was used to calculate the activity coefficients for both cations.

Supplementary Note 3: Time-dependent X-ray reflectivity

3.1. Data analysis. Time-dependent X-ray reflectivity (TXR) measured at two photon energies (ΔE = 0.007 and 0.1 keV) were analyzed using first-order rate equations as

$$R(t) = R_{Rb} \quad \text{when } t < t_{des}$$
$$R(t) = R_{Rb} + (R_{Na}–R_{Rb})\,[1–\exp(–(t–t_{des})/\tau_{App,des})] \quad \text{when } t \geq t_{des} \text{ for Rb}^+ \text{ desorption} \quad (7)$$

$$R(t) = R_{Na} \quad \text{when } t < t_{ads}$$
$$R(t) = R_{Na} – (R_{Na}–R_{Rb})\,[1–\exp(–(t–t_{adas})/\tau_{App,ads})] \quad \text{when } t \geq t_{ads} \text{ for Rb}^+ \text{ adsorption} \quad (8)$$

where R_{Rb} and R_{Na} are reflectivities of the muscovite (001) surface in 3 mM RbCl and 30 mM NaCl solutions at q = 0.488 Å^{-1}, t_{des} and t_{ads} are the time for injection of an exchanging solution for Rb^+ desorption and adsorption, and $\tau_{App,des}$ and $\tau_{App,ads}$ are the apparent time constants for Rb^+ desorption and adsorption (Supplementary Table 1).

3.2. TXR simulation. Time-dependent XR data were simulated at three photon energies near E_o. The calculations were conducted using non-resonant structure factors of the RbCl and NaCl end members derived from the best-fit models determined for the muscovite (001) surface in contact with 3 mM RbCl and 30 mM NaCl solutions, respectively, at q = 0.488 Å^{-1} (Ref. 5), and a resonant anomalous X-ray reflectivity (RAXR) spectrum calculated on the basis of the best-fit models for the RAXR data measured at the muscovite (001) – 3 mM RbCl solution interface at the same q (Ref. 5). The total Rb coverage (θ) and the IS:OS Rb^+ ratio (r) were used as two independent variables. For desorption, the time-dependent variation in θ was expressed as

$$\theta(t/\tau_\theta) = \theta_i[1–\exp(–t/\tau_\theta)] \quad (9)$$

where θ_i is the initial total Rb coverage, i.e., when $t \leq 0$, and τ_θ is the intrinsic time constant for changing the coverage. The ratio r was calculated using time-dependent variations in IS and OS Rb^+ coverages (θ_{IS} and θ_{OS}) as

$$r(t/\tau_r) = \theta_{IS}(t/\tau_r)/\theta_{OS}(t/\tau_r) \quad (10)$$

where τ_r represents the intrinsic time constant for changes in the IS to OS ratio. When $t \geq 0$, $\theta_{IS}(t/\tau_r)$ and $\theta_{OS}(t/\tau_r)$ are calculated as

$$\theta_{IS}(t/\tau_r) = [\theta(t/\tau_\theta)/\theta_i]\,(\theta_{IS})_i[1–\exp(–t/\tau_r)] \quad (11)$$

$$\theta_{OS}(t/\tau_r) = [\theta(t/\tau_\theta)/\theta_i]\,[(\theta_{OS})_i + (\theta_{IS})_i\exp(–t/\tau_r)] \quad (12).$$

The time-dependent variation in non-resonant structure factor, $F_{NR}(t/\tau)$, was calculated using a combination of two methods. The θ-dependent variation in $F_{NR}(t/\tau_\theta)$ was calculated on the basis of the linear combination of predetermined end-member values $F_{NR,RbCl}$ and $F_{NR,NaCl}$ as[3,4]

$$F_{NR}(t/\tau_\theta) = [\theta(t/\tau_\theta)/\theta_i]\cdot F_{NR,RbCl} + (1– [\theta(t/\tau_\theta)/\theta_i])\cdot F_{NR,NaCl} \quad (13)$$

The r-dependent variation was computed by calculating $F_{NR,RbCl}$ using changes in the total electron-density profile by subtracting (for IS Rb^+) and adding (for OS Rb^+) electron densities of Rb^+.

$$F_{NR,RbCl}(t/\tau_r) = F_{NR,RbCl}(0) + (f^o_{Rb} – f^o_{H_2O})\Sigma_j\Delta\theta_j(t/\tau_r)\exp(iqz_j)\exp[–(qu_j)^2/2] \quad (14)$$

where θ_j, z_j, and u_j are the coverage, heights from the surface, and rms width of Rb^+ ion j where j = IS and OS, respectively. f^o_{Rb} and $f^o_{H_2O}$ are the atomic form factors for Rb^+ and water molecules, which were approximated to be the number of electrons (36 and 10) in the calculation for simplicity.

We demonstrate three exemplary cases: (a) when the total Rb^+ coverage decreases while there is no change in IS:OS Rb^+ (i.e., $\tau_\theta = \tau$ and τ_r = infinite), (b) when the total Rb^+ coverage is constant while IS Rb^+ changes to OS Rb^+ (i.e., τ_θ = infinite and $\tau_r = \tau$), and (c) when both the total Rb^+ coverage decreases and IS Rb^+ changes to OS Rb^+ at the same rate (i.e., $\tau_\theta = \tau_r = \tau$).

(a) Case 1: *No change in IS:OS Rb^+ with decreasing total coverage* (Supplementary Fig. 3a). This simulates a situation when Rb^+ desorbs from the muscovite surface without changes in the IS:OS ratio. The temporal variation in the total electron density shows exponential decreases in both IS and OS Rb^+ coverages when $t > 0$ while the RAXR spectra maintain its original shape. Two calculated TXR plots above E_o (by 0.007 and 0.1 keV) show a similar time-dependent trend. The apparent time constants (τ_{app}) derived from fits of the TXR to an exponential decay function for these two cases are close to the intrinsic time constant for reaction (τ) (Supplementary Table 2). The TXR plot below E_o (–0.1 keV) shows negligible intensity variations.

(b) Case 2: *Change from IS to OS Rb^+ with a constant total coverage* (Supplementary Fig. 3b). This simulates a process where adsorbed Rb^+ speciation changes from IS-dominant to OS-dominant types while maintaining a constant total coverage. The time-resolved RAXR (TRAXR) data show a substantial change in spectral shape, which results from a continuous increase in average height of adsorbed Rb^+ at the interface[3]. A significantly larger intensity change is observed for TXR at $E–E_o$ = 0.007 keV than at $E–E_o$ = 0.1 keV (Supplementary Fig. 3b and Supplementary Table 2). The TXR data at $E–E_o$ = –0.1 keV also show a significant change in intensity unlike those for Case 1 which show almost no intensity change. The data analyses show the τ_{app} values at $E–E_o$ = 0.007 and 0.1 keV higher than τ (Supplementary Table 2), indicating that the reaction will appear to be slower in these TXR data.

(c) Case 3: *Change from IS to OS Rb^+ with decreasing total coverage* (Supplementary Fig. 3c). This simulation is similar to what was observed in the actual dataset during Rb^+ desorption (Fig. 2). When t > 0, the coverage of IS Rb^+ decreases in a faster rate than intrinsic desorption rate τ because it shifts to OS Rb^+ before it desorbs from the interface. At the initial stage, the OS Rb^+ coverage increases when $t/\tau \leq \sim 1$ and then decreases. The TRAXR data show the combined effects observed for (a) and (b): the decrease in the signal amplitude near E_o as well as the changes in the spectral shape. The changes in shape lead to different responses in TXR at three E. For example, τ_{app} at $E–E_o$ = 0.007 keV is about 40% smaller than at $E–E_o$ = 0.1 keV (Supplementary Table 2).

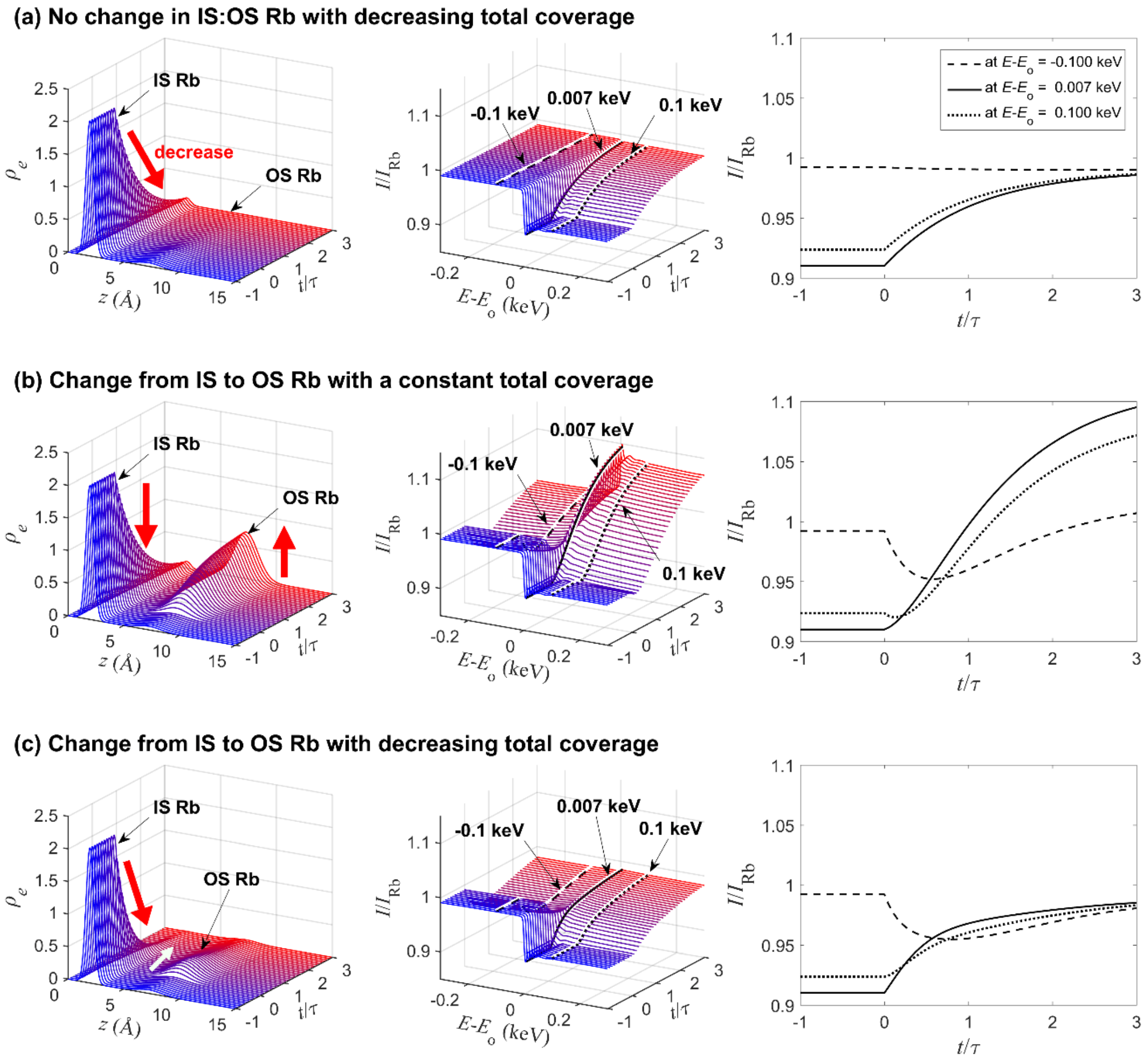

Supplementary Figure 3. Simulated time-dependent variations in the electron-density profile of Rb^+ (ρ_e) as a function of height (z) from the muscovite surface (left), resonant anomalous X-ray reflectivity (middle), and X-ray reflectivity (right) at three photon energies (below, near, and above E_o). The calculations were conducted for three different Cases a–c. See the text for details.

Supplementary Table 1. Apparent time constants for exchange reactions between Rb^+ and Na^+ adsorbed at the muscovite (001) – water interface.

ΔE (keV)	$\tau_{App,des}$ (sec)	$\tau_{App,ads}$ (sec)
0.007	7.1 ± 0.8	7.1 ± 0.5
0.1	15 ± 2	7.6 ± 0.8

Supplementary Table 2. Analyses of three simulated TXR data in Supplementary Figure 3.

Cases	E–E_o (keV)	τ_{app}/τ	$(I/I_{Rb})_i$	$(I/I_{Rb})_f$	$[(I/I_{Rb})_f-(I/I_{Rb})_i]/(I/I_{Rb})_i$
Case 1	0.007	1.03	0.91	0.99	8.0%
	0.1	1.01	0.92	0.99	6.6%
Case 2	0.007	1.24	0.91	1.12	**21%**
	0.1	1.23	0.92	1.09	**17%**
Case 3	0.007	**0.87**	0.91	0.99	8.0%
	0.1	**1.31**	0.92	0.99	6.6%

The calculated TXR data for $t/\tau \geq 0$ were fit to $I/I_{Rb}(t/\tau) = (I/I_{Rb})_i + [(I/I_{Rb})_f - (I/I_{Rb})_i]\times[1-\exp(-(t/\tau)/\tau_{app})]$, where τ_{app} is the apparent time constant, $(I/I_{Rb})_i$ and $(I/I_{Rb})_f$ are initial and final (I/I_{Rb}) values , i.e., when $t/\tau \leq 0$ and $t/\tau = \infty$, during the simulated exchange reactions.

Supplementary Note 4: Model-independent TRAXR analyses

Time-resolved resonant anomalous X-ray reflectivity, $R(q,E,t)$, is proportional to $|F_{tot}(q,E,t)|^2$ where $F_{tot}(q,E,t)$ is the total structure factor. $F_{tot}(q,E,t)$ can be expressed as

$$F_{tot}(q,E,t) = [F_{NR}(q,t) + \{f'(E)+if''(E)\}\, \mathcal{F}_R(q,t)]^2 \qquad (15)$$

where F_{NR} is the time-dependent non-resonant total structure factor, $f'(E)+if''(E)$ is the resonant anomalous dispersion of a specific element (e.g., Rb^+ in our study), and $\mathcal{F}_R(q,t)$ is the time-dependent element-specific partial structure factor of the element[6]. The $F_{NR}(q,t)$ values during solution exchange were calculated using those of the muscovite (001) surface in pure salt solutions (3 mM RbCl and 30 mM NaCl) using equation (13), as described previously[3,4]. The $f'(E)+if''(E)$ value was determined experimentally using the X-ray absorption spectrum measured near E_o in transmission mode through a 0.1 M RbCl solution followed by the differential Kramers-Kronig transform[7].

The TRAXR data were fit using the model-independent approach[6] where $\mathcal{F}_R(q,t)$ is expressed using time-dependent variations in amplitude, $A_R(q,t)$, and phase, $\Phi_R(q,t)$ as

$$\mathcal{F}_R(q,t) = A_R(q,t)\exp[i\Phi_R(q,t)] \qquad (16)$$

This partial structure factor can also be written as the sum of the structure factors of the IS and OS complexes as

$$\mathcal{F}_R(q,t) = \theta_{Rb,IS}(t)\exp(iqz_{Rb,IS})\exp[-(qu_{Rb,IS})^2/2] + \theta_{Rb,OS}(t)\exp(iqz_{Rb,OS})\exp[-(qu_{Rb,OS})^2/2] \qquad (17)$$

using the heights (z) and distribution widths (u) of IS and OS Rb^+ that were predetermined from full structure analyses of the RAXR data measured from the muscovite (001) – 3 mM RbCl solution interface[5].

Supplementary Note 5: Free energy profile of Rb^+ adsorbed at the muscovite mica (001) – water interface

The potential energy profile (Fig. 6) was constructed on the basis of the best-fit model of Rb^+ distribution for the RAXR data measured at the muscovite (001) – 3 mM RbCl solution interface. The Gibbs free energy, ΔG^o_{ads}, as a function of height z is expressed as

$$\Delta G^o_{ads}(z) = –RT\log[\rho_{Rb}(z)/\rho_{Rb}(\infty)] \quad (18)$$

where R is the gas constant, T is the temperature (= 298 K), $\rho_{Rb}(z)$ is the number density profile of Rb^+ expressed in units of molarity derived from the best-fit model, and $\rho_{Rb}(\infty)$ = 3 mM is the bulk Rb^+ concentration. The z-dependent uncertainty of the potential profile, $\sigma\Delta G^o_{ads}(z)$, was calculated by propagating the uncertainty of the number density profile, $\sigma\rho_{Rb}(z)$, as[8]

$$\sigma\Delta G^o_{ads}(z) = –RT\ \sigma\rho_{Rb}(z)/\rho_{Rb}(z). \quad (19)$$

Supplementary Note 6: Apparent coverage calculations

Both the TXR signals at $E–E_o$ = 0.007 and 0.1 keV (Figs. 2b–e) and the adsorbed Rb^+ coverage derived from the TRAXR analyses (Figs. 4c and d) show monotonic variations with reaction time. A comparison between these two quantities shows a well-defined 1:1 relationship between them (Supplementary Fig. 4). This relationship allows us to estimate an “apparent Rb^+ coverage” from a measured XR signal at a specific photon energy. The estimation sequence is described in Supplementary Figure 5.

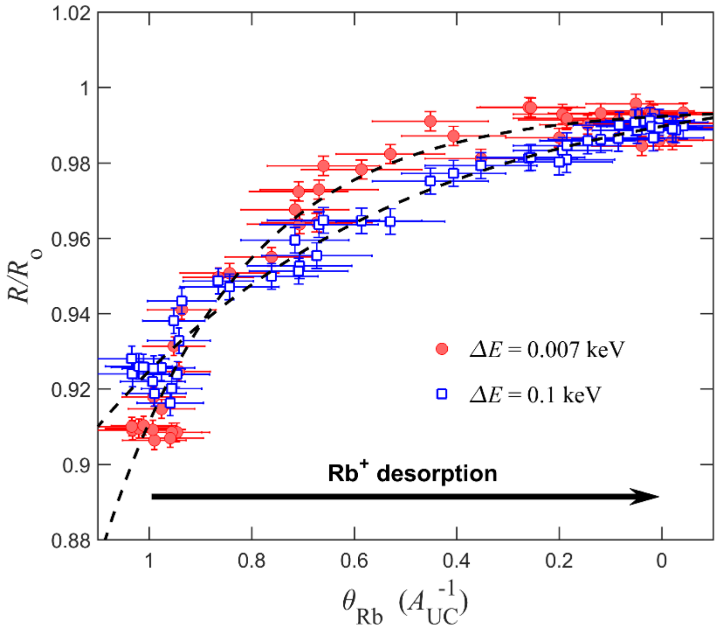

Supplementary Figure 4. 1:1 relationship between X-ray reflectivity and adsorbed Rb^+ coverage during Rb^+/Na^+ exchange at the muscovite (001)–water interface. The reflectivity data (R) were measured during Rb^+ desorption at ΔE = 0.007 and 0.1 keV (Figs. 2b and d of the manuscript). The adsorbed Rb^+ coverage (θ_{Rb}) was derived from the TRAXR analyses (Fig. 4c of the manuscript). The s.d. uncertainties were derived from the counting statistics and non-linear least-squares fits of the data using the model-independent RAXR analyses[6]. X-ray reflectivity (R) is normalized to the non-resonant reflectivity (R_o) calculated using the best-fit model of the non-resonant X-ray reflectivity data for the mica (001) – 3 mM RbCl system[5]. The dashed lines are provided to guide the the eye.

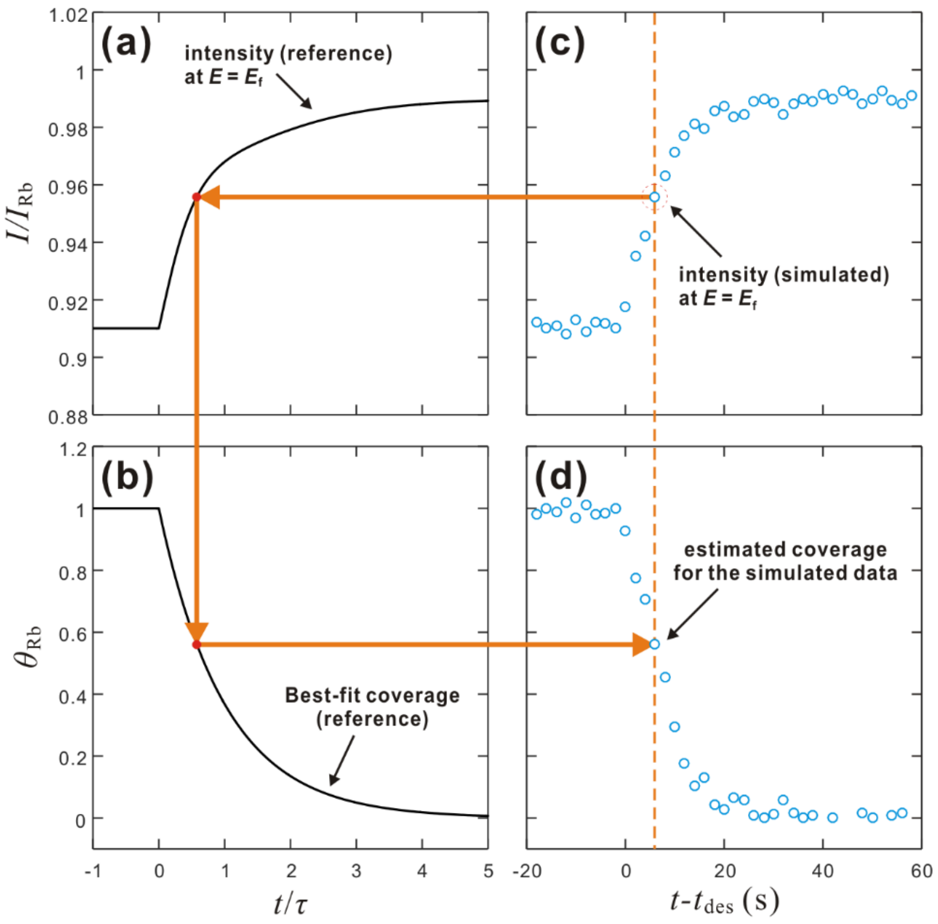

Supplementary Figure 5. Apparent Rb^+ coverage estimation. (a) Reference normalized reflectivity at a fixed photon energy (E = E_f) as a function of time normalized to the time constant (τ). The data are from Supplementary Fig. 3c at E–E_o = 0.007 keV and **(b)** Best-fit coverage of the corresponding reflectivity data. The coverages were calculated from the full TRAXR analyses as shown in Figs. 3 and 4. **(c)** Simulated normalized reflectivity at the same photon energy (E = E_f). **(d)** Apparent Rb^+ coverage estimated for the simulated data. The orange arrows show a sequence of estimating the Rb^+ coverage by comparing the simulated intensity at a time of measurement (t–t_{des} where t_{des} is a time when Rb^+ desorption starts) with the reference intensity plot.

Supplementary References

1. Lee, S.S., Fenter, P., & Park, C., Optimizing a flow-through X-ray transmission cell for studies of temporal and spatial variations of ion distributions at mineral-water interfaces. *J. Synchrotron Radiat.* 20, 125-136 (2013).
2. Langmuir, D., *Aqueous Environmental Geochemistry*. (Prentice-Hall, Inc., Upper Saddle River, NJ, 1997).
3. Park, C., Fenter, P.A., Sturchio, N.C., & Nagy, K.L., Thermodynamics, interfacial structure, and pH hysteresis of Rb^{+} and Sr^{2+} adsorption at the muscovite (001)-solution interface. *Langmuir* 24, 13993-14004 (2008).
4. Lee, S.S., Fenter, P., Nagy, K.L., & Sturchio, N.C., Changes in adsorption free energy and speciation during competitive adsorption between monovalent cations at the muscovite (001)-water interface. *Geochim. Cosmochim. Acta* 123, 416-426 (2013).
5. Lee, S.S., Fenter, P., Nagy, K.L., & Sturchio, N.C., Monovalent ion adsorption at the muscovite (001) - solution interface: Relationships among ion coverage and speciation, interfacial water structure, and substrate relaxation. *Langmuir* 28, 8637-8650 (2012).
6. Park, C. & Fenter, P.A., Phasing of resonant anomalous X-ray reflectivity spectra and direct Fourier synthesis of element-specific partial structures at buried interfaces. *J. Appl. Crystallogr.* 40, 290-301 (2007).
7. Cross, J.O. *et al.*, Inclusion of local structure effects in theoretical X-ray resonant scattering amplitudes using *ab initio* X-ray-absorption spectra calculations. *Phys. Rev. B: Condens. Matter* 58, 11215-11225 (1998).
8. Lee, S.S., Park, C., Fenter, P., Sturchio, N.C., & Nagy, K.L., Competitive adsorption of strontium and fulvic acid at the muscovite-solution interface observed with resonant anomalous X-ray reflectivity. *Geochim. Cosmochim. Acta* 74, 1762-1776 (2010).
